# Insulin glargine effectively achieves glycemic control and improves insulin resistance in patients with early type 2 diabetes that exhibit a high risk for cardiovascular disease

**DOI:** 10.3892/etm.2014.1688

**Published:** 2014-04-24

**Authors:** JILING LI, ZHENGPING FENG, QIFU LI, YAN HE, CHANGHONG ZHAO, JUN HE

**Affiliations:** Department of Endocrinology, The First Affiliated Hospital of Chongqing Medical University, Chongqing 400016, P.R. China

**Keywords:** insulin glargine, type 2 diabetes mellitus, glycemic control, insulin resistance, cardiovascular risk

## Abstract

In the present study, the clinical efficacy and safety of administering insulin glargine to early type 2 diabetes (T2D) mellitus patients with a high risk for cardiovascular disease were assessed. A total of 42 early T2D patients at a high risk for cardiovascular disease were randomly divided into an insulin-glargine group and a standard-care group. The patients in the insulin-glargine group received oral antidiabetic agents plus glargine once a day via a subcutaneous injection. The patients in the standard-care group were administered oral antidiabetic agents according to the diabetic treatment guidelines. The median follow-up period was 6.4 years. Comparisons were made between the two groups with regard to levels of plasma glucose, glycosylated hemoglobin (HbA1c) and plasma lipids, the homeostasis model assessment-insulin secretion index (HOMA-β) and HOMA-insulin resistance index (HOMA-IR), as well as the incidence of hypoglycemia, adverse cardiovascular events and body mass index (BMI). The fasting plasma glucose level in the insulin-glargine group was significantly lower than that observed in the standard-care group. However, the levels of 2-h postprandial glucose, HbA1c and plasma lipids, as well as the BMI, were similar when comparing the two groups. Although the level of the HOMA-β did not differ between the two groups, the level of HOMA-IR in the insulin-glargine group was significantly lower than that observed in the standard-care group. During the follow-up period, the incidence of hypoglycemia in the insulin-glargine group was significantly higher when compared with the standard-care group, however, no significant difference in the incidence of adverse cardiovascular events was observed. Therefore, the results of the present study indicated that insulin glargine may effectively achieve glycemic control and improve insulin resistance without increasing the risk for cardiovascular events in early T2D patients that were considered to be at a high risk for cardiovascular disease.

## Introduction

The prevalence of diabetes mellitus in China is rapidly increasing with the aging population and ~9.7% of the adult population (~92.4 million) have diabetes (1). Furthermore, diabetes has been identified to be an independent risk factor for cardiovascular disease, whereby an elevated fasting plasma glucose (FPG) level is considered to be significant (2,3). In the early stages of type 2 diabetes (T2D), a number of residual β-cells remain, thus, early insulin therapy can improve β-cell function and enhance the control of plasma glucose levels. This reduces glucotoxicity and ultimately reduces or prevents the development and progression of diabetes-associated cardiovascular complications (4,5).

The American Diabetes Association and the European Association for the Study of Diabetes emphasized the importance of basal insulin treatment in newly diagnosed diabetes patients in 2009 (6). However, few studies have been performed investigating whether basal insulin therapy decreases cardiovascular events in patients with early T2D at a high risk for cardiovascular disease. In addition, a limited number of studies have investigated whether insulin glargine improves β-cell function and insulin sensitivity in T2D patients. Therefore, the aim of the present study was to investigate whether insulin glargine was able to reduce the risk of cardiovascular events and improve β-cell function and insulin sensitivity in T2D patients with a high risk for cardiovascular disease. Furthermore, the long-term efficacy and safety of insulin glargine were also evaluated.

## Patients and methods

### Patients

In total, 42 patients (in- or outpatients; males, 17; females, 25; age, ≥50 years) who had recently been diagnosed with T2D mellitus and were considered to be at a high risk for cardiovascular disease were included in the present study. The patients were randomly divided into an insulin-glargine group (n=22) and standard-care group (n=20). Patients were diagnosed with a high risk for cardiovascular disease if they exhibited any one of the following symptoms: i) History of myocardial infarction, stroke or revascularization; ii) angina with documented ischemic changes; iii) albuminuria; iv) left ventricular hypertrophy identified by electrocardiogram or echocardiogram; v) stenosis of ≥50% in the coronary, carotid or lower extremity arteries; and vi) ankle/brachial index of <0.9. Patients were excluded if they exhibited diabetic ketoacidosis, hyperosmolar nonketotic hyperglycemic coma or marked hepatorenal damage. The present study was approved by the Ethics Committee of The First Affiliated Hospital of Chongqing Medical University (Chongqing, China) and written informed consent was obtained from all the participants.

Subjects in the insulin-glargine group received a subcutaneous injection of insulin glargine at an initial dose of 10 U/day as well as their current glycemic-control regimen (not including thiazolidinediones). The dose of glargine was adjusted based on the FPG level, targeting a self-measured FPG level of ≤5.3 mmol/l. Subjects in the standard-care group were administered oral antidiabetic agents, and if necessary, insulin (not including glargine) was also administered according to the diabetic treatment guidelines. The target was to obtain an FPG level of ≤6.1 mmol/l and a 2-h postprandial blood glucose (2hPG) level of ≤8.0 mmol/l. Other drugs administered to the participants remained unchanged throughout the follow-up. The patients were assessed every 3–6 months and the median follow-up period was 6.4 years. Levels of plasma glucose, glycosylated hemoglobin (HbA1c) and plasma lipids were measured and recorded at each follow-up. Patients’ weight was measured annually for calculation of the body mass index (BMI). At the final follow-up examination, the levels of plasma insulin and C-peptide were detected and the homeostasis model assessment-insulin resistance index (HOMA-IR) and the HOMA-insulin secretion index (HOMA-β) were calculated as follows: HOMA-IR = fasting plasma insulin × FPG/22.5; and HOMA-β = 20 × fasting plasma insulin/(FPG - 3.5). In addition, the incidence of hypoglycemia and adverse cardiovascular events, including cardiovascular fatality, coronary heart disease, non-fatal myocardial infarction, angina, stroke, revascularization and heart failure, were recorded.

### Glucose oxidase assay

Plasma glucose levels were measured using the glucose oxidase method. Briefly, 0.02 ml distilled water, 0.02 ml glucose standard solution and 0.02 ml test serum were added to three tubes (blank, standard and assay tubes), respectively. A mixed reagent of enzyme and phenol (3 ml) was added to each tube and mixed thoroughly by shaking. Subsequently, the three tubes were placed into a water bath at 37°C for 15 min. The blank tube was used to adjust the instrument to zero and the absorbance values of the standard and assay tubes were measured at a wavelength of 505 nm on an automatic analyzer (Model 7600, Hitachi High-Technologies Corporation, Ibaraki Prefecture, Japan). The concentration of plasma glucose was calculated using the following formula: Serum glucose concentration (mmol/l) = 5 × (assay tube absorbance/standard tube absorbance). Each sample was analyzed three times and the average values were recorded.

### High performance liquid chromatography

HbA1c concentration was measured using high performance liquid chromatography. Whole blood samples, that had been obtained from the patients and refrigerated at 4°C, were mixed thoroughly and the concentration of HbA1c was determined using an automatic HbA1c analyzer (Bio-Rad D10; Bio-Rad, Hercules, CA, USA), according to the manufacturer’s instructions. Each sample was assessed three times and the average values were recorded.

### Chemiluminescence assay

A chemiluminescence assay was conducted to determine the plasma insulin and C-peptide levels. Reagents that had been refrigerated at 4°C, were placed into test plates and mixed for 15 min. A calibrating solution and control serum were added to the test plates for the purposes of calibration and quality control. The blood samples were centrifuged at 999 × g for 10 min and the supernatants were transferred to sample plates and labeled for the assay. Each sample was analyzed three times and the average values were recorded. The samples were analyzed by an automated chemiluminescent immunoassay analyzer (ADVIA Centaur, Bayer, Leverkusen, Germany).

### Automatic biochemical analysis

Plasma lipid levels were assessed using an automatic biochemical analyzer. Patient blood samples were centrifuged at 999 × g for 10 min and the supernatants were analyzed to determine the content of total cholesterol, triglycerides and high density and low density lipoproteins, according to the manufacturer’s instructions. Each sample was assessed three times and the average values were recorded.

### Statistical analysis

Statistical analysis was performed using SPSS 17.0 software (SPSS, Inc., Chicago, IL, USA) and the normally distributed and continuous variables are presented as the mean ± standard deviation. Variations from the baseline values and intergroup comparisons were analyzed using the Student’s t-test (paired and unpaired, respectively). HOMA-β and HOMA-IR values were compared between the two groups using the Student’s t-test following logarithmic transformation. The Wilcoxon rank sum test was used for intergroup comparisons of non-normally distributed variables, including the incidence of hypoglycemia and cardiovascular events. Comparisons of plasma insulin and C-peptide levels between the two groups were conducted using repeated measures design analysis of variance. P<0.05 was considered to indicate a statistically significant difference.

## Results

### Insulin glargine treatment reduces the level of FPG

**Figure 1:**
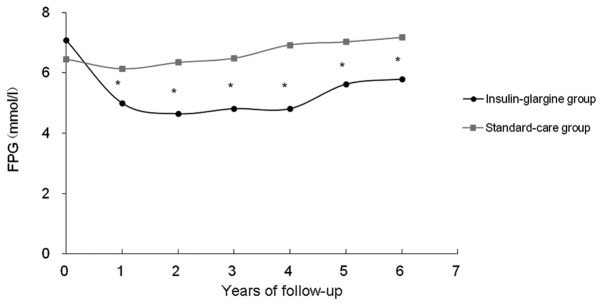
Changes in the FPG level. Outpatients were followed-up every 3–6 months to determine the FPG levels using a glucose oxidase assay. Following treatment, the mean FPG level in the insulin-glargine group demonstrated a constant overall reduction from 7.07 to 5.79 mmol/l (P<0.01) during the 6.4-year treatment period. The FPG level in the insulin-glargine group was significantly lower than that observed in the standard-care group during the follow-up period. ^*^P<0.05, vs. standard-care group. FPG, fasting plasma glucose.

**Figure 2:**
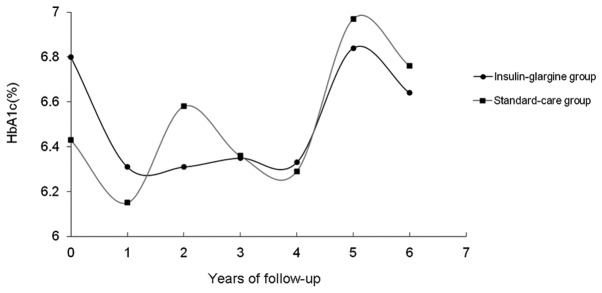
Changes in the HbA1c level. Outpatients were followed-up every 3–6 months to assess the HbA1c levels using high performance liquid chromatography. Following treatment, the mean HbA1c level in the insulin-glargine group did not significantly change during the 6.4-year treatment period. In addition, the levels of HbA1c did not differ between the two groups. HbA1c, glycosylated hemoglobin.

**Figure 3:**
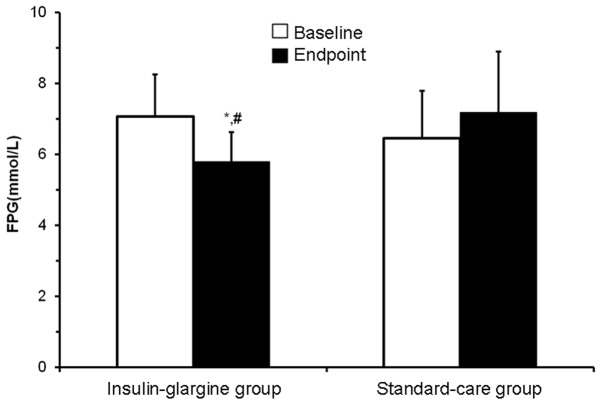
Changes in the FPG levels in the two groups between the baseline and the study endpoint. FPG levels were determined at the beginning of the study and at the final follow-up examination using a glucose oxidase assay. The mean FPG level in the insulin-glargine group changed significantly between the baseline and the endpoint. ^*^P<0.01, vs. baseline; ^#^P<0.05, vs. standard-care group. FPG, fasting plasma glucose.

The baseline characteristics of the subjects are shown in Table I. Overall, the baseline demographics were considered to be relatively uniform between the two groups (P>0.05). To measure the levels of FPG, HbA1c and 2hPG, a glucose oxidase assay and high performance liquid chromatography were conducted. Following treatment, the mean FPG level in the insulin-glargine group demonstrated a constant overall reduction from 7.07 to 5.79 mmol/l over the 6.4-year treatment period (P<0.01; Fig. 1), however, the mean HbA1c level did not alter significantly (Table II and Fig. 2). By contrast, the FPG and HbA1c levels in the standard-care group did not indicate a significant difference prior to and following treatment (Figs. 1 and 2). Through comparing the data at the endpoints between the two groups, it was identified that the FPG level in the insulin-glargine group (5.79±0.83 mmol/l) was significantly lower than the level in the standard-care group (7.17±1.77 mmol/l; P<0.05), however, the levels of HbA1c and 2hPG did not differ between the two groups (Table III and Fig. 3). In addition, the FPG level in the insulin-glargine group was significantly lower than the level observed in the standard-care group during the follow-up period (P<0.05; Table II and Fig. 1). These observations indicated that insulin glargine treatment influenced the reduction in FPG levels, but exhibited no effect on the levels of HbA1c or 2hPG.

### Insulin glargine treatment affected the levels of plasma insulin and C-peptide in the initial stages and reduced the level of HOMA-IR, but not HOMA-β

To determine the levels of plasma insulin and C-peptide, a chemiluminescence assay was performed. On completion of the study, the levels of plasma insulin and C-peptide at fasting and at 30 min following oral glucose tolerance test (OGTT) in the insulin-glargine group were significantly lower than those observed in the standard-care group (P<0.05), however, there were no statistically significant differences identified between the two groups at 60 and 120 min following OGTT. In addition, the HOMA-IR value in the insulin-glargine group was significantly lower compared with the standard-care group (P<0.01), whereas no statistically significant difference was observed between the two groups with regard to HOMA-β (Table IV). These observations indicated that the insulin glargine treatment affected the levels of plasma insulin and C-peptide in the initial stages, which reduced the level of HOMA-IR, but not that of HOMA-β.

### Insulin glargine treatment may result in hypoglycemia, but not adverse cardiovascular events

To investigate the effect of insulin glargine treatment on the incidence of hypoglycemia and adverse cardiovascular events, the patients were closely followed-up throughout the 6.4 years of treatment. The incidences of hypoglycemia in the insulin-glargine and standard-care groups were 11.7 episodes per 100 persons/year (seven individuals with a total of 16 episodes) and 0.8 episodes per 100 persons/year (one individual with one episode), respectively, which was identified to be a statistically significant difference (P<0.05). By contrast, the incidences of adverse cardiovascular events did not differ between the two groups with 4.4 episodes per 100 persons/year in the insulin-glargine group and 11.3 episodes per 100 persons/year in the standard-care group (Table V). These observations indicated that insulin glargine treatment may lead to hypoglycemia.

### Insulin glargine treatment does not affect the levels of plasma lipids or the BMI

To assess the levels of plasma lipids, an automatic biochemical analyzer was employed. The levels of plasma lipids in the two groups did not change significantly from the baseline and the difference between the two groups at the endpoint was not identified to be statistically significant. Between the start of the study and completion, patients’ BMIs increased by 0.15±1.95 kg/m^2^ in the insulin-glargine group and 0.20±1.80 kg/m^2^ in the standard-care group (Table VI), however, analysis between the two groups did not identify a statistically significant difference. These results indicated that insulin glargine treatment did not affect the plasma lipid levels or the BMI.

## Discussion

T2D mellitus is characterized by insulin resistance and the impaired function of β-cells. Through the application of insulin therapy at the initial stages of T2D mellitus to improve the control of plasma glucose levels, it may be possible to reverse the damage on β cells, which results from hyperglycemia (7). In addition, an increased risk for cardiovascular disease in T2D mellitus patients has been observed. Previous studies (8,9), both foreign and domestic, have indicated that the levels of FPG and HbA1c are closely associated with the development and progression of cardiovascular events, and the cardiovascular risk of patients with T2D mellitus may be reduced by the early administration of insulin to attain or approach the normal plasma glucose level.

Insulin glargine is a long-acting insulin analog that can be produced via recombinant DNA technology. Insulin glargine functions slowly and requires a long time to reduce the plasma glucose level, without exhibiting a peak value and simulates the physiological secretion of basal insulin (10,11). In the present study, the FPG level in the insulin-glargine group significantly decreased from the baseline values, and the long-term FPG and HbA1c concentrations were maintained at near-normal levels. Furthermore, following treatment, the FPG level in the insulin-glargine group was significantly decreased when compared with the level in the standard-care group. These observations are consistent with the results of previous studies (12,13).

β-cell function in T2D mellitus patients is known to progressively deteriorate. Therefore, previous studies have assessed whether the early administration of insulin to improve glucose control may result in improved insulin resistance and β-cell function. Pistrosch *et al* (14) demonstrated that glargine improved β-cell function and insulin resistance in newly diagnosed T2D mellitus patients. However, the present study indicated that there was no statistically significant difference in the level of HOMA-β between the two groups. By contrast, the level of HOMA-IR in the insulin-glargine group was significantly lower when compared with the standard-care group. Although the insulin secretion conditions of each participant were not measured on entry into the study, we may hypothesize that insulin glargine treatment improves the insulin resistance of patients with T2D mellitus; this hypothesis is consistent with previous studies (15,16). The underlying mechanism may be that the early administration of glargine reduces the damage to β cells and target organs that is caused by high plasma glucose levels, which activates the insulin signaling pathway and improves insulin resistance. However, this specific mechanisms requires further investigation.

Previous studies (17,18) have demonstrated a low incidence of hypoglycemia in T2D mellitus patients that have been treated with insulin glargine. By contrast, the results of the present study indicated that there were more hypoglycemic episodes in the insulin-glargine group when compared with the standard-care group. This result may have been observed since the FPG level in the insulin-glargine group was required to be ≤5.3 mmol/l, which was associated with an increased insulin glargine dose and therefore an increased risk of hypoglycemia.

T2D mellitus patients are considered to be at a greater risk of cardiovascular disease. Holman *et al* (19) demonstrated that insulin treatment on recently diagnosed T2D mellitus patients resulted in the improved control of plasma glucose levels, which in turn reduced the risk of cardiovascular events. By contrast, several large-scale studies (20–23) have indicated that hypoglycemia induced by intensive glucose-lowering therapy, is strongly associated with the development of cardiovascular diseases in patients with T2D mellitus. The results of the present study demonstrated that during the intervention period, the incidence of hypoglycemia was significantly higher in the insulin-glargine group as compared with the standard-care group, however, the risk of cardiovascular events was similar between the two groups. There are a number of possible explanations for this result. Firstly, the relatively higher risk of hypoglycemia in the insulin-glargine group may have resulted in an increased risk of cardiovascular disease, which may marginally offset the protective mechanism of glargine on the cardiovascular system. Secondly, all the participants exhibited a high risk for cardiovascular diseases, therefore, the benefit of glargine on the cardiovascular system in these subjects was less likely to be observed as compared with T2D mellitus patients that were without cardiovascular risks. Finally, the antihypertensive agents, lipid-modulating agents and anticoagulants that exhibit beneficial effects on the cardiovascular system were continued throughout the treatment period, thus, to a certain extent, the cardiovascular benefit of insulin glargine was difficult to observe. Therefore, interpretation of the results indicates that glargine may reduce the incidence of cardiovascular events should the follow-up period be extended.

In conclusion, insulin glargine treatment results in favorable outcomes with regard to long-term glycemic control and the improvement of insulin resistance, without increasing the risk of cardiovascular events in patients with T2D mellitus. The observations of the present study indicate that glargine may be considered as an effective and safe basal insulin in clinical applications.

## Figures and Tables

**Table I:** Baseline demographic characteristics of the subjects.

Variable	Insulin-glargine group (n=22)	Standard-care group (n=20)
Age (years)	62.8±6.3	62.7±5.8
Male:female	10:12	7:13
BMI (kg/m^2^)	24.32±2.51	24.90±2.78
FPG (mmol/l)	7.07±1.18	6.45±1.36
HbA1c (%)	6.80±0.79	6.43±1.13
TC (mmol/l)	4.71±0.96	4.82±1.28
TG (mmol/l)	1.51±1.03	1.87±1.68
HDL (mmol/l)	1.15±0.22	1.22±0.30
LDL (mmol/l)	2.78±0.72	2.79±1.04

BMI, body mass index; FPG, fasting plasma glucose; HbAlc, glycosylated hemoglobin; TC, total cholesterol; TG, triglyceride; HDL, high-density lipoprotein; LDL, low-density lipoprotein.

**Table II:** Glycemic indices during the trial.

	FPG (mmol/l)		HbAlc (%)	
		
Follow-up	Insulin-glargine group (n=22)	Standard-care group (n=20)	Insulin-glargine group (n=22)	Standard-care group (n=20)
Baseline	7.07±1.18	6.45±1.36	6.80±0.79	6.43±1.13
Year 1	4.99±0.82^a^	6.13±0.97	6.31±0.59	6.15±0.64
Year 2	4.64±0.84^a^	6.34±1.07	6.31±0.70	6.58±1.00
Year 3	4.81±0.78^a^	6.48±1.25	6.35±0.78	6.36±1.01
Year 4	4.81±0.82^a^	6.92±1.23	6.33±0.74	6.29±0.84
Year 5	5.62±0.96^a^	7.02±1.63	6.84±0.80	6.97±0.94
Year 6	5.79±0.83^a^	7.17±1.77	6.64±0.81	6.76±1.15

a P<0.05, vs. standard-care group.

FPG, fasting plasma glucose; HbAlc, glycosylated hemoglobin.

**Table III:** FPG and HbA1c levels on completion of the trial.

Variable	Insulin-glargine group (n=22)	Standard-care group (n=20)
FPG (mmol/l)	5.79±0.83^a^^b^	7.17±1.77
HbA1c (%)	6.64±0.81	6.76±1.15

a P<0.05, vs. standard-care group;

b P<0.01, vs. baseline.

FPG, fasting plasma glucose; HbA1c, glycosylated hemoglobin.

**Table IV:** Levels of plasma insulin and C-peptide on completion of the trial.

Plasma level	Insulin-glargine group (n=22)	Standard-care group (n=20)
FCP (ng/ml)	1.67±1.01^c^	2.59±1.13
30′ CP (ng/ml)	3.31±1.82^c^	4.84±1.87
60′ CP (ng/ml)	5.25±2.07	6.21±2.42
120′ CP (ng/ml)	6.97±2.62	8.41±3.27
FINS (mIU/l)	8.47±4.08^c^	11.12±2.99
30′ INS (mIU/l)	18.03±8.36^c^	23.43±6.64
60′ INS (mIU/l)	27.07±11.31	29.69±8.68
120′ INS (mIU/l)	36.97±14.03	42.34±10.06
HOMA-β^a^	77.37±26.80	80.76±61.56
HOMA-IR^b^	2.56±1.32^d^	3.54±1.33

a 20 × FINS/(FPG - 3.5);

b FINS × FPG/22.5.

c P<0.05 and

d P<0.01, vs. standard-care group.

FCP, fasting C-peptide; CP, C-peptide; FINS, fasting plasma insulin; INS, plasma insulin; HOMA-β, homeostasis model assessment insulin secretion index; HOMA-IR, homeostasis model assessment insulin resistance index.

**Table V:** Incidence of hypoglycemia and adverse cardiovascular events throughout the study.

Variable	Insulin-glargine group (n=22)	Standard-care group (n=20)
Hypoglycemia, n (n/100 persons/year)^a^	16 (11.7)^c^	1 (0.8)
Cardiovascular events, n (n/100 persons/year)^b^	6 (4.4)	14 (11.3)

a This category included any episode of hypoglycemia for which the patients required assistance (confirmed by a self-measured plasma glucose level of ≤3.9 mmol/l) or from which the patients recovered promptly following oral intake of carbohydrates.

b Cardiovascular events included cardiovascular mortality, coronary heart disease, non-fatal myocardial infarction, angina, stroke, revascularization and heart failure.

c P<0.05, vs. standard-care group.

**Table VI:** Changes in patient BMI and levels of plasma lipids at the baseline and endpoint.

	Insulin-glargine group (n=22)		Standard-care group (n=20)	
		
Variable	Baseline	Endpoint	Baseline	Endpoint
BMI (kg/m^2^)	24.32±2.51	24.47±2.12	24.90±2.78	25.10±2.62
TC (mmol/l)	04.71±0.96	04.47±0.89	04.82±1.28	04.54±0.85
TG (mmol/l)	01.51±1.03	01.42±0.79	01.87±1.68	01.85±1.07
HDL (mmol/l)	01.15±0.22	01.23±0.21	01.22±0.30	01.33±0.31
LDL (mmol/l)	02.78±0.72	02.65±0.74	02.79±1.04	02.54±0.68

BMI, body mass index; TC, total cholesterol; TG, triglyceride; HDL, high-density lipoprotein; LDL, low-density lipoprotein.
